# The transverse-vertical incision (Alazzam hybrid incision)

**DOI:** 10.1007/s00423-021-02404-5

**Published:** 2022-01-23

**Authors:** Moiad Alazzam, Mostafa Abdallah Khalifa, Abdallah Al-Ani

**Affiliations:** 1 Consultant Gynaecological Oncology Surgeon, Department of Gynaecological Oncology, Churchill Hospital-Oxford University Hospitals, Old Road, Oxford, Headington OX3 7LN UK; 2 Department of Gynaecological Oncology, Fellow in Gynaecological Oncology, Oxford, UK; 3 School of Medicine, Jordan University, Amman, Jordan

**Keywords:** Vertical incision,, Transverse incision,, Maylard,, Midline incision,, Abdominopelvic surgery

## Abstract

Open abdominal surgery evolved around two incisions, vertical and transverse incisions. Transverse incisions are associated with less postoperative morbidities but offer limited access. Vertical incisions offer better access but are associated with more complications. We describe here a hybrid incision, transverse-vertical incision that offers adequate exposure for complex lower abdominopelvic surgery while overcoming the limitations and morbidities associated with midline and transverse incisions.

## Background

Abdominal surgery is an essential component for healthcare and requires a significant proportion of hospital resources. Although laparoscopy/robotic procedures are becoming more popular, still a significant proportion, particularly the larger, more complex procedures, are performed by open technique[1]. Surgical access to the abdomen and pelvis can be achieved through multiple incision types, which can be broadly divided into either midline, including paramedian, or transverse, including oblique[2].

The type of abdominal incision can influence multiple outcomes. In practice, the choice of incision is usually based on the surgeon’s preference rather than the patient’s criteria. For the surgeon, ease of access, time to open and close the abdomen, and incidence of postoperative complications such as hernia and delayed recovery are important. For the patient, pain, cosmetic appearances, and rapid return to normal function are important. Economically the duration of operation and duration of hospital stay determine cost [2, 3].

**Fig. 1:**
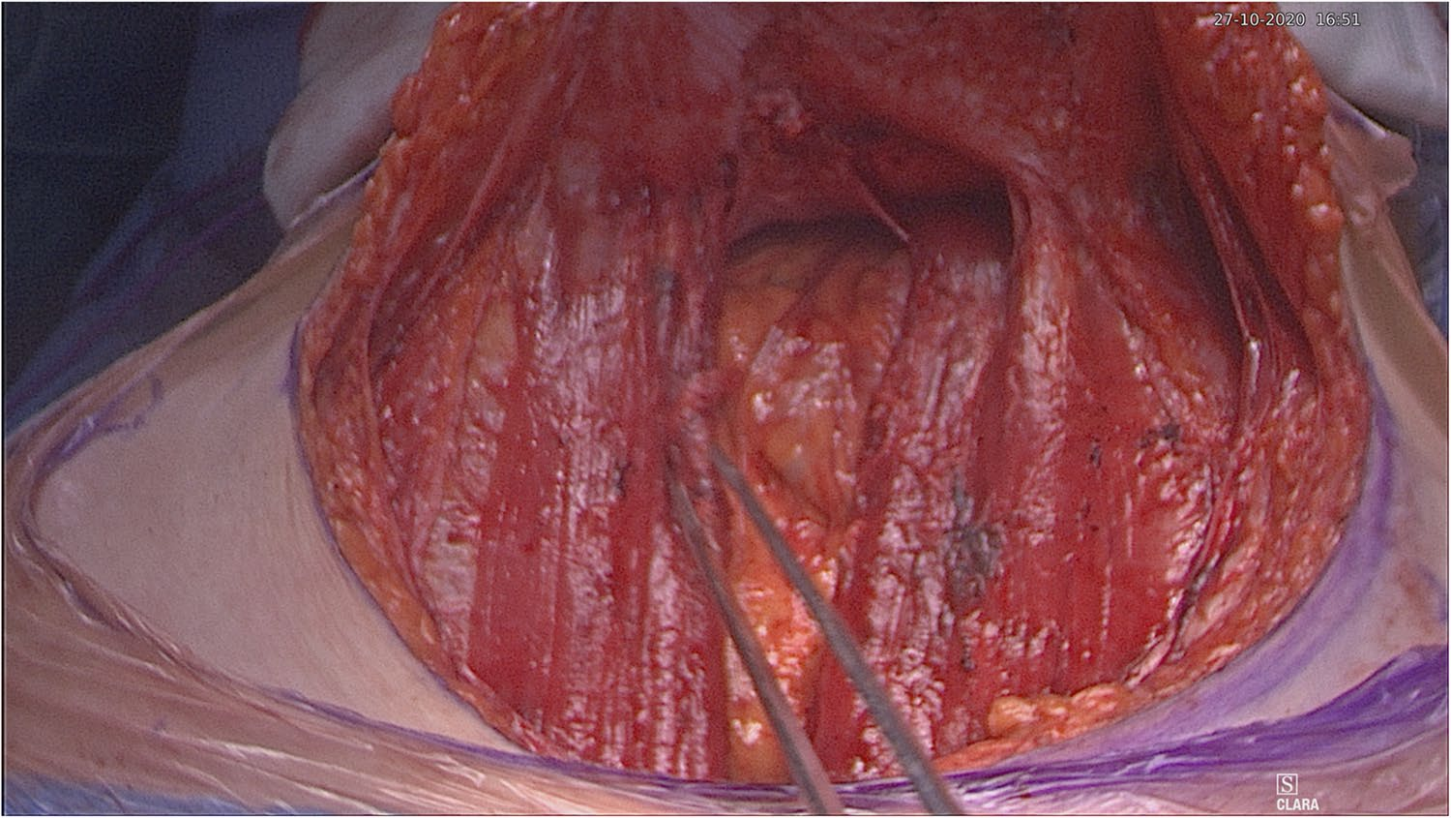
General view of the incision; landmarks, transverse, and vertical incisions

The publication of the LACC trial intensified the debate of what is the best surgical approach for women diagnosed with early-stage cervical cancer[4]. Herein, we describe a step-by-step hybrid transverse-vertical incision, Alazzam hybrid incision which retains the benefits of both transverse and midline incisions (Fig. 1). To our knowledge, this incision has not been described to date.

## Alazzam hybrid incision—step by step

**Fig. 2:**
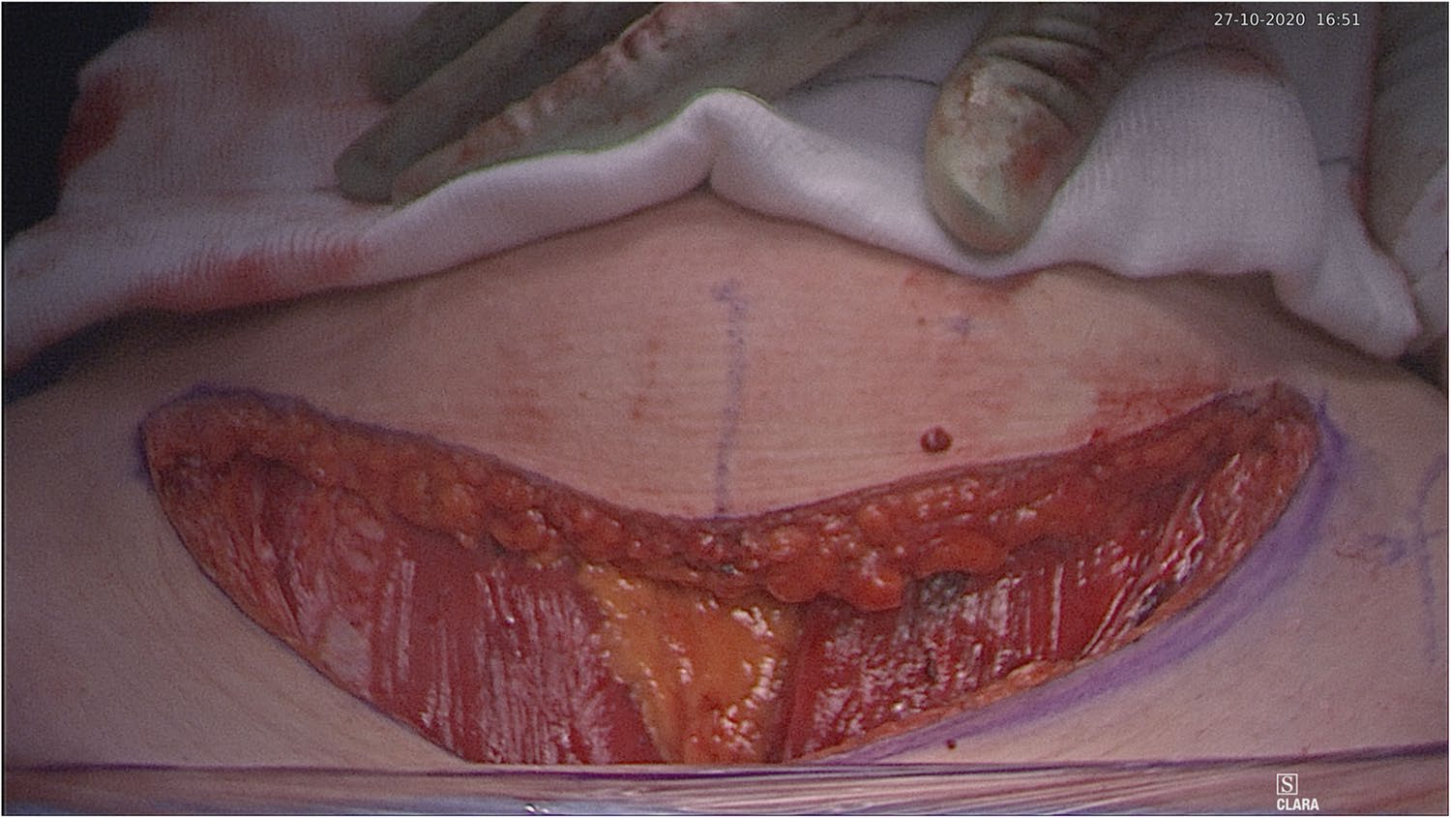
Skin and bone landmarks

**Fig. 3:**
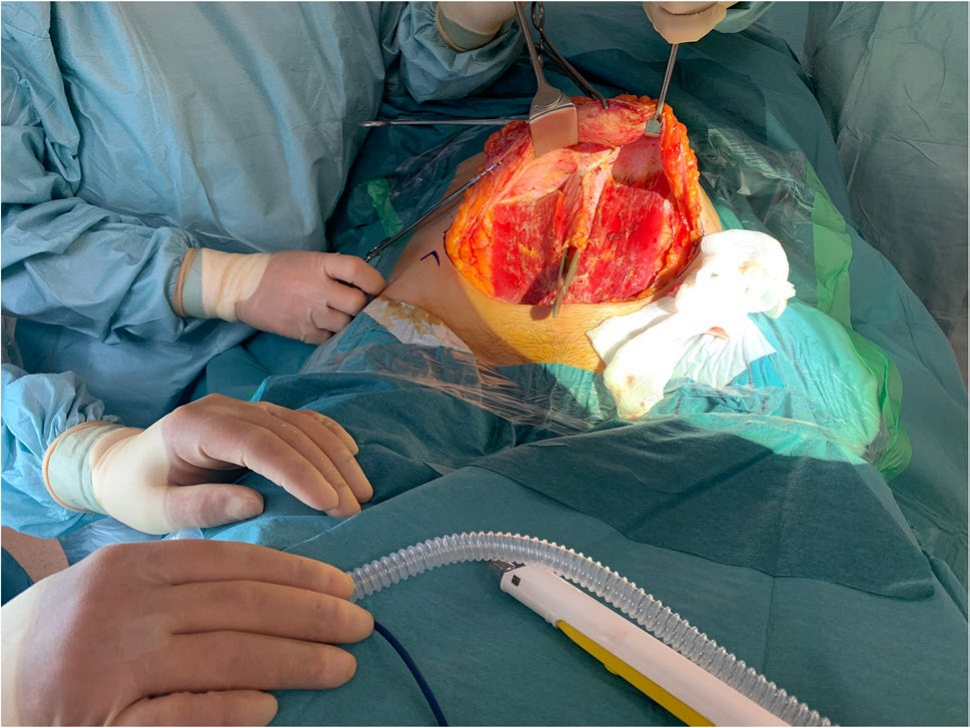
Exposure after completion of anterior rectus sheath leaf transverse dissection

**Fig. 4:**
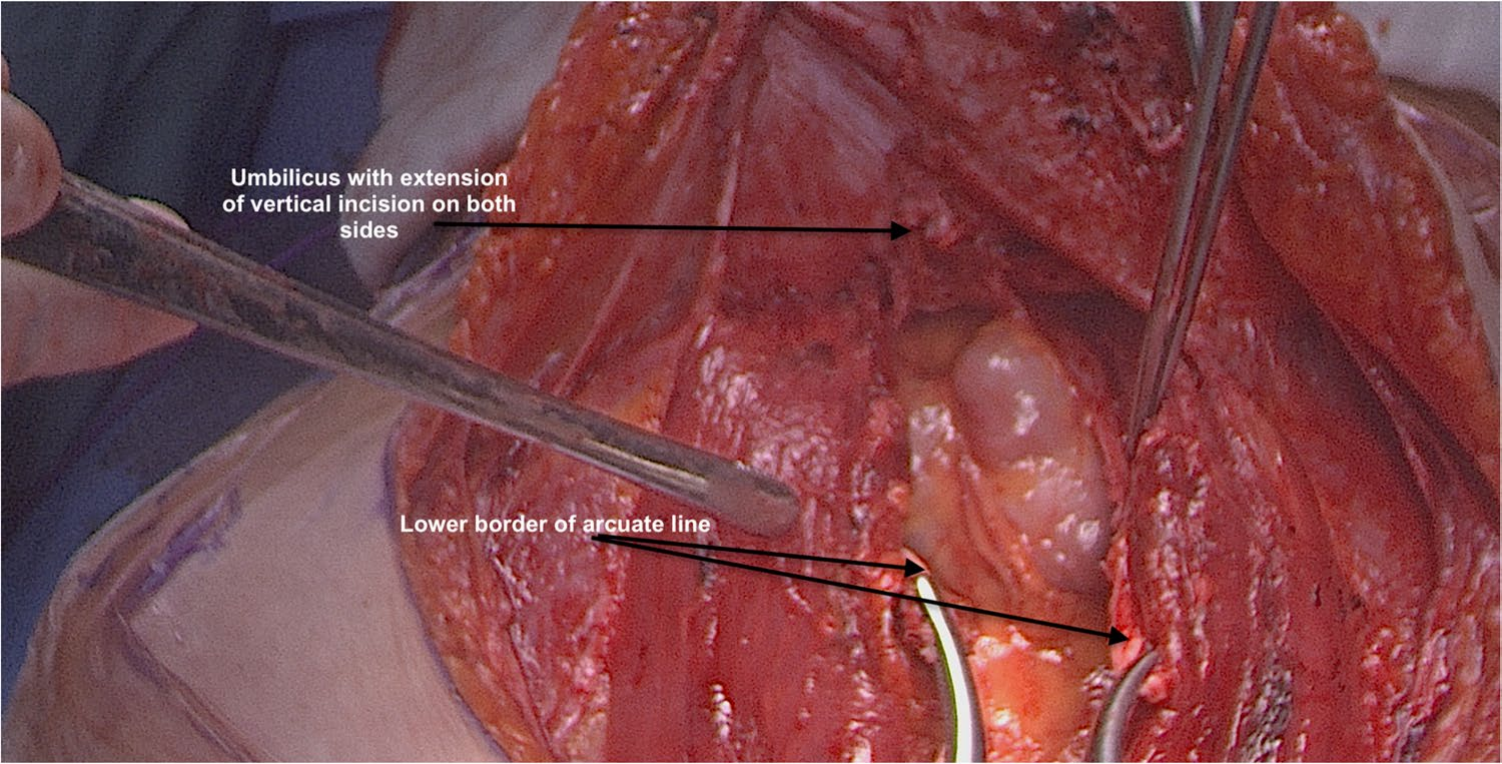
Exposure after completion of posterior rectus sheath leaf vertical dissection

**Fig. 5:**
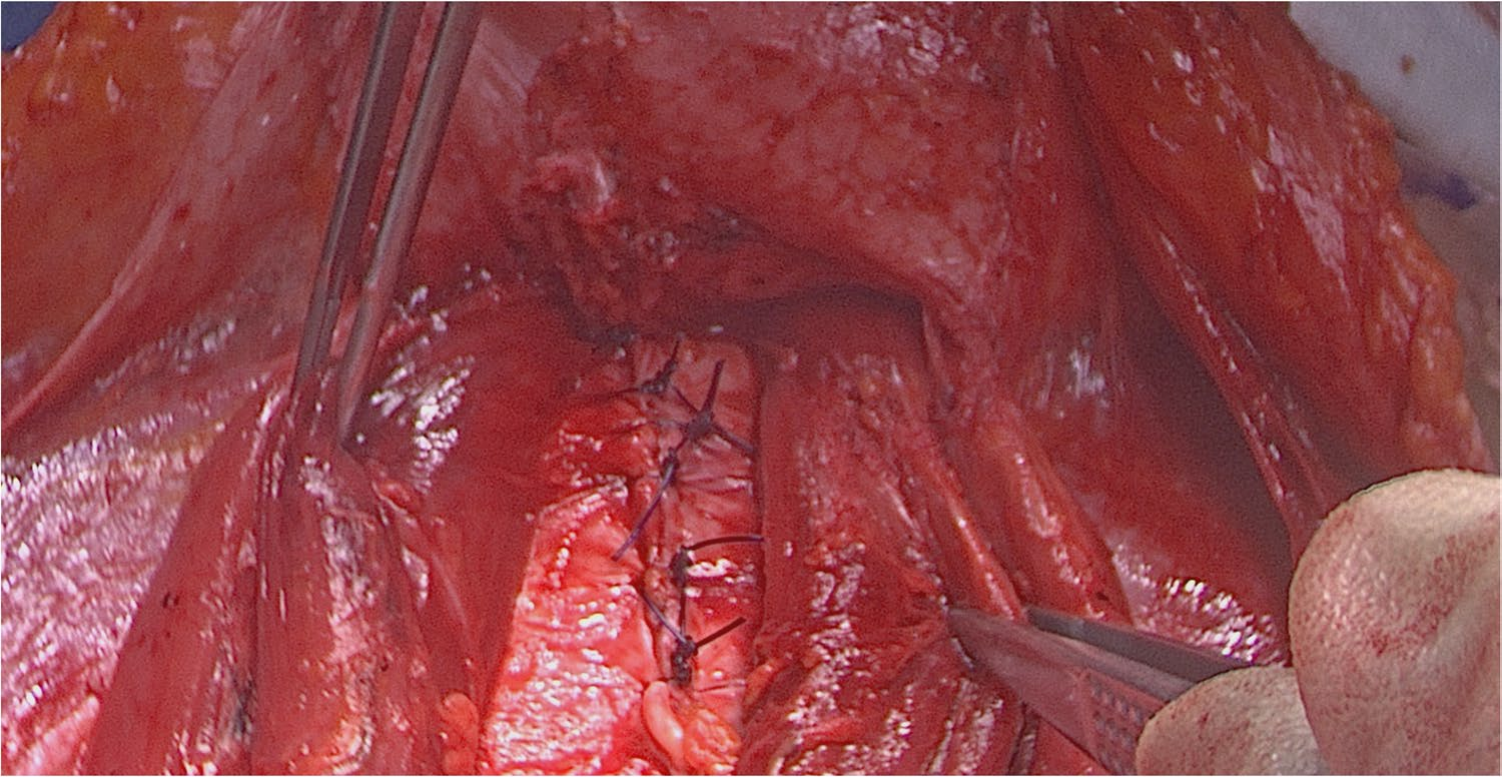
Closure of rectus sheath posterior leaf

**Fig. 6:**
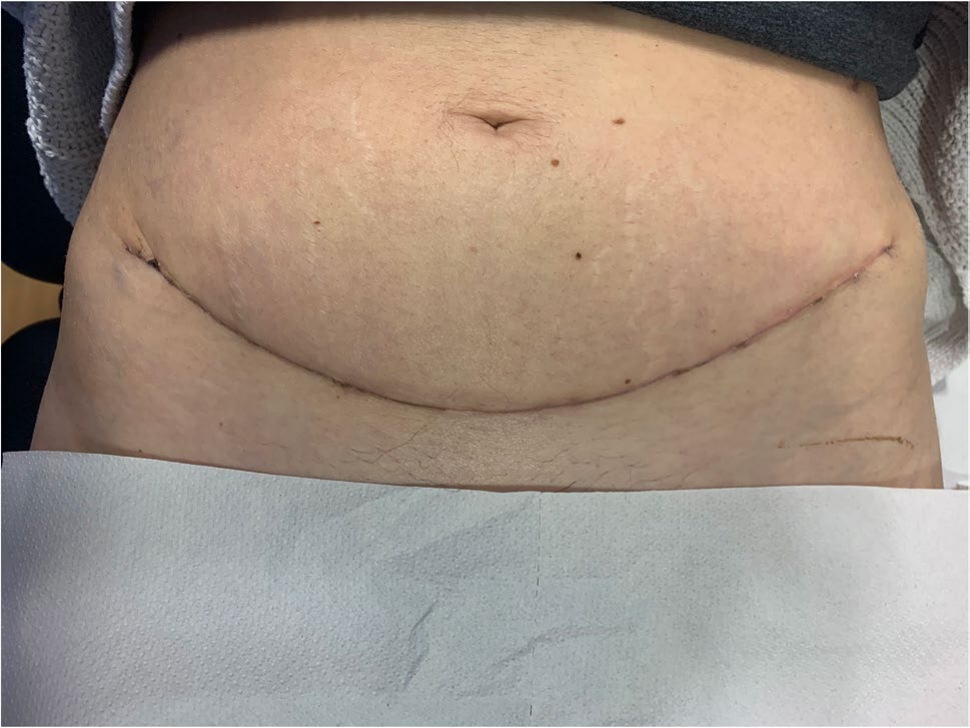
Appearance of skin 4 weeks post-surgery

The patient is placed in a modified lithotomy position. After cleaning the abdomen and draping the patient, the abdomen is opened using an Alazzam hybrid incision as described below:

1. Identification of anatomical landmarks (Fig. 2)
  - Upper lateral incision borders: a mark is made 2 cm medio-cephalad to the anterior superior iliac spine (ASIS) on each side.
  - A third mark is made 3–5 cm in the midline above the upper border of the symphysis pubis.
  - A curved line is drawn with the convexity toward symphysis pubis with a curve of a small radius circle.
2. Incision for the outer layers (Fig. 3)
  - Skin is incised along the curved line drawn either with cutting diathermy needle or surgical scalpel and fat layer opened in a routine manner.
  - The outer leaf of the rectus sheath is opened (transversely) mirroring the shape of the skin incision.
  - The anterior surface of the rectus muscles carefully dissected off the posterior wall of the outer rectus sheath leaf up to the level of the umbilicus centrally and to the level of the incision margins laterally.
  - The rectus muscles carefully dissected off the anterior surface of the inner rectus sheath leaf extending up to 2 cm above the level of the umbilicus
3. Vertical incision (Fig. 4)
  - The inner rectus sheath layer (midline) is divided starting from the arcuate ligament and extending up to the umbilicus.
  - If further extension is needed, then the inner rectus sheath can be divided on either side of the umbilicus.
4. Closure (Fig. 5)
  - The inner leaf of the rectus sheath is closed with interrupted monofilament suture (we use PDS #1). We normally start from the upper angle until the arcuate line. (Fig. 5).
  - The outer leaf of the rectus sheath is closed transversally with continuous suture using Loop PDS with alternating simple interrupted sutures to decrease tension and risk of hernia.
  - Skin is normally closed subcutaneous with 3/0 Monocryl (Figs. 6 4 weeks post-surgery).

**Fig. 7:**
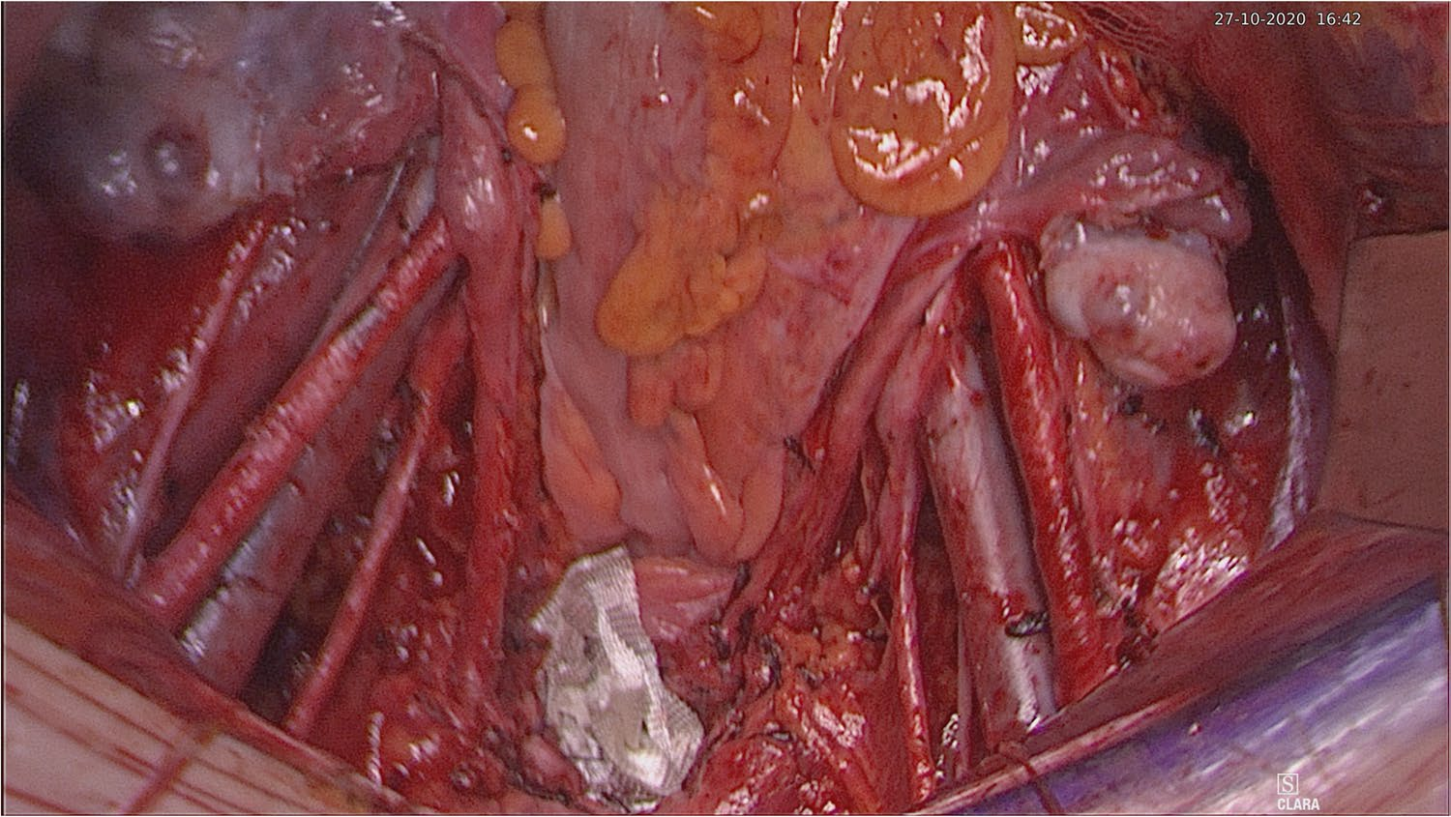
Adequacy of operating surgical field exposure

## Methods and materials

Following the publication of the LACC trial in 2018 and the subsequent changes to surgical approach in particular cervical cancer, Alazzam hybrid incision was introduced in mid-2020 with the aim to overcome the potential morbidities from the midline and Maylard incisions.

We prospectively recorded the data for all patients who underwent radical pelvic surgery using “Alazzam hybrid incision”. Extracted data included age, BMI, total operating time, intraoperative complications, immediate and delayed postoperative complications, use of analgesia, and incidence of hernia.

Between May 2020 and April 2021 (Table 1); 12 patients underwent primary radical pelvic surgery using Alazzam hybrid incision, 11 had stage 1 cervical cancer, two patients had radical trachelectomy, and nine radical abdominal hysterectomies. Patients were followed up until October 2021. The 12th patient had the surgery for high-grade endometrial cancer. In all patients, bilateral pelvic lymphadenectomy was performed. The surgery was completed successfully in all patients without any need for extension or midline conversion. None of the patients had any significant intraoperative complications. The average blood loss was 409 ml. Postoperatively, the pain was well controlled—paracetamol only (*n* = 4), paracetamol, and as required codeine or ibuprofen (*n* = 8). There was no reported hernia in all patients. There were no reported long-term complications during the follow-up period.

**Table 1:** Study cohort outcomes using Alazzam hybrid incision

Characteristics	AHI (*n* = 12)
Body mass index, median (IQR)	29.14 (8.7)
Estimated blood loss, median (IQR)	409 (317)
Length of surgery (min), median (IQR)	296.3 (36.1)
Hospital stay (day), median(IQR)	5 (1)
Conversion to midline	0
Ureteric injury	0
Accidental bladder/bowel injury	0
Hernia	0
Admission to ITU	0
Return to theatre < 24 h	0

## Discussion

The success of abdominopelvic surgeries is dependent on a variety of factors, including the type of incision, site of incision, adequacy of exposure, and optimal closure[5]. Furthermore, surgery is increasingly being utilized in high-risk patients with multiple concomitant comorbidities [6]. Therefore, it is of vital importance to consider the safety of procedures while ensuring the overall efficacy in terms of postoperative recoveries, such as immediate pain relief and/or pulmonary function.

The choice of incision is contingent on a myriad of factors, including the adequacy of site exposure, dissemination pattern of malignancy, presence of extra-pelvic metastasis, presence of obesity, and patient’s cosmetic considerations [7]. Albeit, the choice of incision is frequently based on the surgeon’s own preference and expertise rather than any patient or economic considerations [2]. Throughout the literature, abdominopelvic surgeries were reported to be conducted using three different types of incisions including midline vertical incisions, suprapubic transverse incisions (i.e., Pfannenstiel, Maylard, and Cherney), and infra/supraumbilical incisions [8], each of which is associated with a different profile of surgical advantages and postoperative complications.

Midline incisions are almost exclusively used in gynecologic cancer surgery as it allows quick entry into the abdominal cavity with minimal blood loss and can be easily extended depending on intraoperative findings [9]. However, these incisions are associated with inadequate cosmetic results and are coupled with complications such as wound dehiscence, incisional hernias, and pulmonary deterioration [10–12]. These complications may result from the presence of an avascular wound bed which hinders wound healing, and the burden of tension on wound closure resulting from the contralateral contraction of abdominal muscles perpendicular to the incision’s direction[12, 13]. Due to the perpendicular nature of vertical/midline incisions with reference to the oblique muscle layer, it cuts medial to they might be associated with more pain due to nerve damage as nerves run in a parallel fashion to the oblique muscle layer crossing the midline. Bickenbach et al. (2013) conducted a meta-analysis on all randomized trials reporting on incision types and demonstrated significantly higher narcotic use in patients undergoing midline incisions further fortifying the aforementioned statements [12].

On the other hand, transverse techniques are associated with superior outcomes in terms of cosmetics, wound healing, the incidence rate of incisional hernia, wound strength, and interferes less with postoperative respiration [7, 9, 12]. However, their disadvantages include intense hemorrhage, abdominal nerve injury, and is more time consuming [7]. Transverse incisions’ greatest limitation is its limited exposure into the abdominal cavity, which renders the entire incisional technique as a second-choice modality, particularly in radical hysterectomies and pelvic lymph node dissections [10]. While both the Cherney and Maylard incisions act as a feasible alternative to midline laparotomy due to increased pelvic exposure [14], the Cherney incision is twice as fast with an average completion time of 1 to 2 min, spares the inferior epigastric resulting in less bleeding and hematoma formation, and is muscle “separating” as it cuts parallel to the rectus muscle fibers providing greater wound strength [3]. On the other hand, Maylard incisions, despite being muscle cutting, retain the advanced of transverse incisions and are considered a suitable alternative to laparoscopy and in women with cervical cancer and complex pelvic conditions due to its excellent exposure of the pelvic sidewalls [15].

The Alazzam hybrid incision retains the advantages associated with both transverse and midline incisions while avoiding their inherent limitations. The incision is a muscle separating procedure that does not attempt to ligate the inferior epigastric vessels. Therefore, it retains the benefits of a Cherney incision, being muscle separating, while its avoidance of manipulating the inferior epigastric vessels contributes to less bleeding, less hematoma and neuroma formation, and maintains the vascularity of the rectus muscle and the wound bed underneath. Similar to a Pfannenstiel incision, the Alazzam hybrid incision does not impact pulmonary function and results in an optimal cosmetic result. Due to its muscle splitting nature, the Alazzam hybrid incision contributes to less pain on deep breathing, which enables it to avoid affecting the patient’s ventilatory capacity, as historically documented [16, 17]. Moreover, the incision does not require a steep learning curve retaining both pace and operational simplicity compared to laparoscopic techniques which require experience and technological setup [7, 18]. The Alazzam hybrid incision’s greatest strength and potential lie in its ability to provide adequate exposure, comparable to that of midline incisions, to the abdominopelvic cavity without any major compromises in terms of neither morbidity nor mortality. Therefore, the incision is suitable for pelvic and lower abdominal procedures (Table 2) (Figs. 6 and 7).

**Table 2:** Summary the various common incisions

Name of the incision	Measurement	Muscle cutting	Advantages	Disadvantages
Midline (median) incision	Can be extended depending on required exposure	No	Excellent exposure Easily extendable Minimum nerve damage Rapid entry to abdomen	Pain Hernia Poor cosmetic outcome
Paramedian incision	Can be extended depending on required exposure	Yes	Same as median incision	Higher infection rates Hemorrhage Longer operative time
Pfannenstiel incision	10–15 cm long and 2 cm above the pubic symphysis	No (can be used to widen the incision)	Better cosmetic appearance Less pain Less interference with postoperative respirations Greater strength	Limited access to upper abdomen and pelvic sidewall Hematomas Poor exposure
Joel‐Cohen incision	10–12 cm long 3–5 cm above pubic symphysis	No	Same as Pfannenstiel	Limited access to upper abdomen and pelvic sidewall Hematomas Poor exposure
Cherney incision	2 cm above the umbilicus. Can extend to the level of anterior superior iliac spine	Yes (a tendon detaching incision)	Same as Pfannenstiel Access to pelvic sidewall	Hematomas (lower risk than Maylard) Myonecrosis Osteomyelitis Limited access to upper abdomen
Maylard incision	4 cm above the symphysis pubis and extended laterally until 3 cm from the anterior superior iliac spine	Yes	Same as Pfannenstiel Access to pelvic sidewall	Impaired circulation in the lower extremity Hematomas Limited access to upper abdomen
Alazzam hybrid incision (described in this paper)	3 cm above symphysis pubis extending laterally to 2 cm medial and above ASIS	No	Same as Pfannenstiel and low midline incision Access to pelvic sidewall and abdomen below kidney	Time to enter abdomen (not suitable for emergency) Limited access to upper abdomen

Nonetheless, the Alazzam hybrid incision’s most significant limitation lies in its time to completion. The technique incorporates two different incisional types at different tissue levels of the abdominopelvic plane. Moreover, appropriate suturing and wound closure at two consecutive perpendicular angles partially contribute to increasing the overall length of the entire operation. Such limitation may theoretically predispose the technique to be associated with more complications, including but not limited to wound infections, surgical site infections, or venous thrombosis [19]. However, such theoretical risk is highly unlikely to be of significance since it would require the incision to prolong the duration of operation in a magnitude of hours and not mere minutes.

## Conclusion

The evidence generated by the Laparoscopic Approach to Cervical Cancer (LACC) trial demonstrates that minimally invasive surgery results in lower rates of survival in patients with early-stage cervical cancer compared to its open abdominal counterpart [4]. As a result, a thorough understanding of different incision types or the development of superior techniques is of utmost importance considering the evidence-based return of open techniques for cervical cancer. While midline incisions are preferred in emergency and exploratory surgeries due to their ease, speed, and excellent exposure, they are associated with significant morbidity. The Alazzam hybrid incision presents itself as a feasible alternative to midline incisions as they provide surgeons, irrespective of the level of experience, with a fast and functional technique with minimal postoperative morbidity.

## Data Availability

The authors confirm they will provide data if required.
